# Anti-Inflammatory Effects of Cold Thermal Therapy on Allergic Skin Inflammation Induced by Trimellitic Anhydride in BALB/c Mice

**DOI:** 10.1155/2019/1936769

**Published:** 2019-01-28

**Authors:** Chun Hua Piao, Minjoo Kim, Thi Tho Bui, Eunjin Hyeon, Yanjing Fan, Chang Ho Song, Hwan-Jeong Jeong, Ok Hee Chai

**Affiliations:** ^1^Department of Anatomy, Chonbuk National University Medical School, Jeonju, Jeonbuk 54896, Republic of Korea; ^2^Department of Nuclear Medicine, Molecular Imaging and Therapeutic Medicine Research Center, Research Institute of Clinical Medicine, Chonbuk National University Medical School and Hospital, Jeonju, Jeonbuk 54896, Republic of Korea; ^3^Faculty of Biology & Environmental Science, University of Education, The University of Danang, Da Nang 59000, Vietnam; ^4^Institute for Medical Sciences, Chonbuk National University, Jeonju, Jeonbuk 54896, Republic of Korea; ^5^Biomedical Research Institute, Chonbuk National University Hospital, Jeonju, Jeonbuk 54896, Republic of Korea

## Abstract

Cold and hot thermal therapies are widely used as a traditional therapy in many cultures and are often prescribed in the treatment of various musculoskeletal and neurological conditions which present themselves to primary care physicians. However, there are no reports that investigated either the effects of cold and hot thermal therapies on the skin inflammation of trimellitic anhydride- (TMA-) induced dermatitis-like contact hypersensitivity (CHS) mouse model, or the mechanism of thermal therapy on allergic skin inflammation. Therefore, in this study, to reveal the anti-inflammatory effect of thermal therapy and its mechanism on TMA-induced CHS, we analyzed ear-swelling response (ear edema), vascular permeability, serum IgE levels, histological examination, and histamine and Th2 cytokine levels. Cold thermal therapy reduced the ear-swelling response, the vascular permeability, the serum IgE levels, and the infiltration of eosinophils and mast cells as well as the mast cell degranulation. To determine the mechanism by which cold thermal therapy inhibits allergic skin inflammation, detailed studies were carried out revealing that cold thermal therapy suppressed IL-4 and IL-5 secretion and mast cell activation. These results indicated that cold thermal therapy cures skin inflammation of TMA-induced CHS by decreasing Th2 cytokine release, especially IL-4 and IL-5, and mast cell activation. These data suggest that new insight into the mechanism of robust therapeutic effects of cold thermal therapy against allergic dermatitis, and cold thermal therapy may prove to be a useful therapeutic modality on allergic inflammatory diseases as traditional use as well as Th2- or mast cell-mediated allergic responses.

## 1. Introduction

Atopic dermatitis (AD) is a complex and multifactorial chronic inflammatory skin disease that affects up to 18% of children and up to 5% of adults worldwide, with up to 90% of patients presenting with mild to moderate disease [1, 2]. This disease is characterized by erythematous and eczematous lesion, intense pruritus, dryness, and hypersensitive skin. All AD conditions are characterized by elevated peripheral eosinophilia counts and increased serum immunoglobulin E (IgE) levels [2–4]. The patient's skin with AD is very sensitive and has an appearance of chronic redness, after which the skin will thicken, gradually becoming harsh, affecting their appearance and contributing significant psychological, social, and quality-of-life burdens to patients [1–4].

Among the various types of allergic dermatitis, allergic contact dermatitis is a form of contact dermatitis that is induced by an allergic response to a multitude of chemical substances brought on by environmental contamination (allergens) [5–8]. It is well known that the balance of type 1 T helper (Th1) cell/type 2 T helper (Th2) cell cytokines and regulatory T cell/type 17 T helper (Th17) cell cytokines is a very important factor for the pathogenesis of allergic diseases such as allergic dermatitis [8–11]. In addition, local secretion of cytokines, such as TNF-*α*, IL-4, and IFN-*γ*, is very important for the induction of the allergic contact dermatitis [8, 12–14]. In the trimellitic anhydride- (TMA-) induced dermatitis-like contact hypersensitivity (CHS), the repeated challenges of TMA induced biphasic ear-swelling response and early- and late-phase responses [13]. At the repeatedly TMA-induced skin site, the morphologic changes such as eosinophil infiltration and blood vessel dilation are characterized by the delayed type CHS in humans [15–17]. Also, increased vascular permeability leads to dermal and epidermal edema, vascular compaction, and plasma extravasation [18].

Thermotherapy and cryotherapy are widely used as a traditional therapy in many cultures and are often prescribed in the treatment of various musculoskeletal and neurological conditions which present themselves to primary care physicians [19–23]. High-temperature thermal therapy is currently being implemented as a minimally invasive alternative to traditional surgery in the treatment of benign disease and cancer, as well as repair of sports injuries and tissue reshaping or modification [19]. And the use of local heat (thermotherapy) may provide relief of pain and painful muscle spasm through the acceleration of metabolic processes whereby the concentration of pain-inducing toxic metabolites is reduced [21]. Local cooling (cryotherapy) is often more effective in providing pain relief, especially in an acute condition. It acts primarily by decreasing metabolic activity, thus leading to a reduction in inflammatory response, as well as to a decrease in nociceptor excitability, nerve conduction velocity, and muscle contractility [21, 24]. However, these reports are not concentrated on their action mechanism but on just the physiological mechanism including relief of pain, stiffness, and muscle spasm.

The thermal energy can bring physical or biological changes to the tissues, each having a different effect depending on the range of energy applied. The temperature range from 0 to 25°C is shown to decrease blood perfusion and cellular metabolism effects. The temperature range from 40 to 46°C is shown to increase perfusion and induce thermotolerance effects [25–27]. The cold therapy and thermal therapy, using the thermal effects seen in the tissues, are widely used in the clinical area. However, it is reported that tissue damage occurs when tissue temperature is maintained at 15°C for a long period of time [27] and that there is a feeling of discomfort at 43°C [28]. Therefore, the application range was set from 15 to 43°C considering the effectiveness and safety of temperature therapy in this study.

Until now, there are no reports that investigated the effects of cold and hot thermal therapies on the skin inflammation of TMA-induced CHS mouse model, or the mechanism of thermal therapy on allergic skin inflammation. Therefore, in this study, to reveal the anti-inflammatory effect of thermal therapy and its mechanism on TMA-induced CHS, we analyzed ear thickness, serum IgE levels, histological examination, and cytokine levels.

## 2. Materials and Methods

### 2.1. Cold and Hot Thermal Energy Transmitter

In this study, we manufactured and used a device that could generate thermal stimulation (hot and cold). The device comprises a thermal generator, controller, and energy transmission part, and the thermal generator consists of heating and cooling modules. This device is designed to transmit cold and hot energy to the target on a repeated basis. It is capable of controlling the generation of heating and cooling characteristics by setting the operation time and objective temperature.

**Figure 1:**
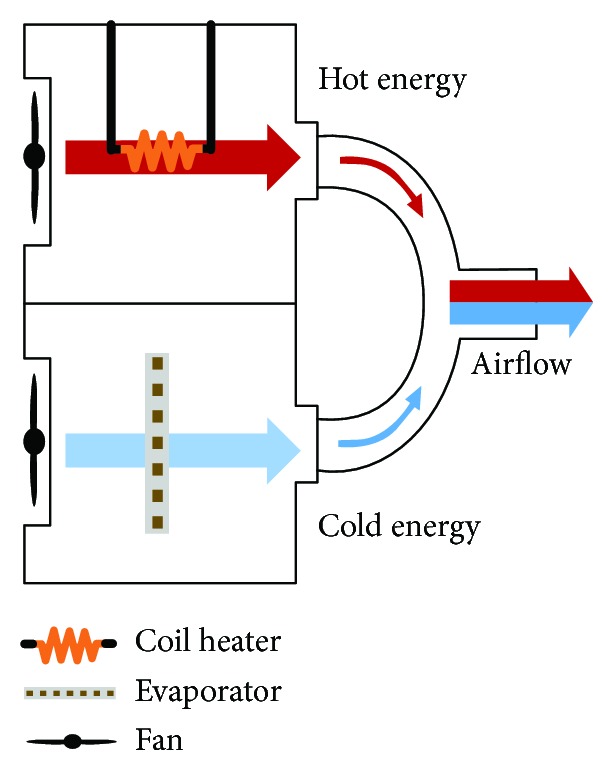
Thermal energy transmit diagram.

The cooling generator consists of the fan (36 × 36 mm, 24 VDC, 0.04 A) and evaporative cooler (self-production), and the heating generator consists of the fan and coil heater (self-production). The thermal energy created in each generator is transferred to the target using the air generated by the internal fans (Figure 1). The temperature of the thermal energy, which is generated in each thermal generator, is measured by the thermal sensor and is transmitted to the control system. The control system operates the cooling or heating generator based on the transmitted temperature.

### 2.2. Animals

Specific pathogen-free male 5-week-old BALB/c mice, weighing approximately 20 g, were purchased from Damool Science (Daejeon, Korea). They were housed 6 per cage in a laminar airflow cabinet maintained at 23 ± 2°C at a relative humidity of 55 ± 10% with a 12-hour dark/light cycle throughout the study. All animal experiments were performed in accordance with the Guidelines for Animal Care and Use and were approved by the Institutional Animal Care and Use Committee of Chonbuk National University Laboratory Animal Center (CBN 2016-37).

### 2.3. Chemicals

Trimellitic anhydride (TMA) was purchased from Sigma-Aldrich (St. Louis, MO, USA) and was dissolved in a mixture of acetone (Junsei, Tokyo, Japan) and olive oil (Filippo Berio, Lucca, Italy) (A/O; vehicle 4 : 1) immediately before application. Formamide solution and Evans blue were purchased from Junsei (Tokyo, Japan) and Sigma-Aldrich (St. Louis, MO, USA), respectively.

### 2.4. Experimental Groups

The mice were randomly divided into five experimental groups of six animals as follows: the vehicle group as a negative control, the TMA group as a positive control, the TMA + hot thermal therapy group (hot, 41°C), the TMA + cold thermal therapy group (cold, 15°C), and the TMA + alternating cold/hot thermal therapy group (alternating, 15/41°C).

### 2.5. Induction of Contact Hypersensitivity (CHS)

**Figure 2:**
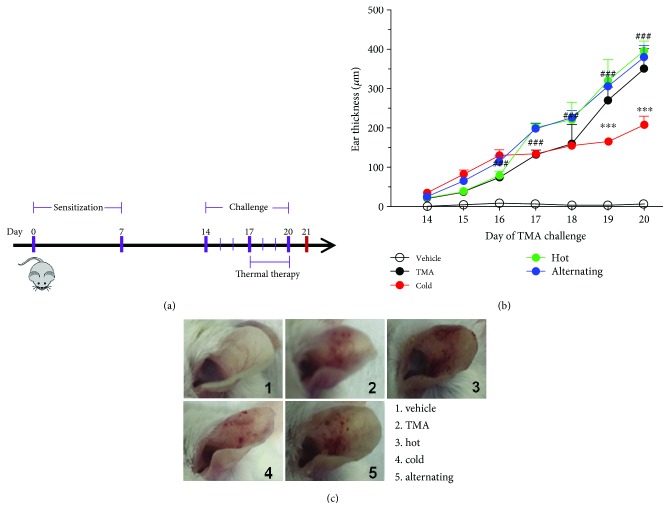
Experimental protocol and inflammation parameter for trimellitic anhydride- (TMA-) induced allergic response in BALB/c mice. (a) Experimental protocol. (b) Ear thickness and (c) photographs were taken after TMA challenges. Data are shown as the mean ± SEM. Statistical comparisons were performed using one-way ANOVA, followed by the Fisher test. Significant differences at ^##^*P* < 0.05 and ^###^*P* < 0.001 compared with the TMA-induced CHS group.

Mice were sensitized on shaved back skin with 100 *μ*l of 50 mg/ml TMA in acetone: olive oil solution (A/O; vehicle) on days 0 and 7 under light anesthesia according to the modified method of Chai et al. From days 14 to 20, each left ear was repeatedly challenged with 20 *μ*l of 50 mg/ml TMA in A/O, and each right ear was repeatedly challenged with 20 *μ*l of A/O. From days 17 to 20, the mice were exposed to high or low thermal energy for 30 minutes and mixed thermal energy for 2 hr every consecutive day. Mice were sacrificed 24 h after the last challenge for further analysis (Figure 2(a)).

### 2.6. Ear-Swelling Measurement

The ear thickness just before and after each TMA challenge was measured three times with a dial thickness gauge (Model 7326, Mitutoyo Manufacturing, Tokyo, Japan), and the difference was defined as ear swelling and expressed in units of 10-4 inches (mean ± SEM). In the time-course study, the ear thickness was measured after each TMA challenge.

Ear swelling was calculated as the following formula:

(1) $$\begin{array}{c} Ear\;swelling=\left[Thickness\text{ of }ear\;after\;TMA\;challenge-Thickness\text{ of }ear\;before\;TMA\;challenge\right]. \end{array}$$

### 2.7. Passive Cutaneous Anaphylaxis (PCA) Reaction

Intravenous dye administration as a means for measuring allergic responses is a versatile assay as it can be used for measuring active, passive, and reverse passive reactions. A solution of 0.5% Evans blue is currently the standard dye used for measuring vascular permeability. To access the allergic responses with PCA in ear tissue, mice were intravenously injected with saline containing 0.5% Evans blue dye (EBD) via the tail vein of the mouse before the last TMA challenge. The EBD was extracted from the ears by incubating the same size ears with 1 ml of formamide overnight at 60°C. Collected perfusion fluid samples were centrifuged (1000 × g, 10 min, at 4°C) at 100 *μ*l each sample; supernatants were obtained to detect the observance at 620 nm using a microplate reader. The vascular permeability of ear tissue was evaluated by calculating the concentration of EBD in each sample according to a standard curve generated from known amounts of EBD.

### 2.8. Measurement of Serum IgE Concentration

24 h after the last TMA challenge, blood was collected from the orbital venous plexus of anesthetized mice 24 h after the last challenge. The samples were centrifuged (1000 × g, 10 min, 4°C) to isolate the serum and stored at -80°C until analysis. IgE level in the serum was measured by enzyme-linked immunosorbent assay (ELISA) kit (R&D Systems Inc., MN, USA).

### 2.9. Cytokine Assay

After sacrifice, 24 h after the last TMA challenge, ear tissues of mice were collected and homogenized in a saline to a concentration of 100 mg/ml with a cOmplete, Mini, EDTA-Free Protease Inhibitor (Roche Applied Science), and the debris-free supernatant was used for cytokine measurement. The levels of IL-4, IL-5, IL-13, and in-ear homogenates were measured by ELISA kits (R&D Systems Inc., MN, USA) according to the manufacturer's protocol.

### 2.10. Histological Examination

24 hours after the last challenge, animals were sacrificed and both ears of the animals were excised. Specimens were fixed immediately by immersion in 10% neutral-buffered formalin solution for 12 hours at 4°C. The fixed specimens were dehydrated in a graded series of alcohols, embedded in paraffin. The sections were cut to 4 *μ*m by a microtome (SM 2000R, Leica, Jena, Germany) and stained with hematoxylin and eosin for general histological structure, Congo red for eosinophil infiltration, and toluidine blue for mast cell activation.

### 2.11. Statistical Analysis

Each experiment was repeated three times with 6 animals per group. Data are expressed as mean ± SEMs. Statistical comparisons were performed using one-way ANOVA, followed by the Fisher test. Statistical significance was defined as *P* < 0.05.

## 3. Results

### 3.1. Cold and Hot Thermal Energy Transmitter

The device successfully produced cold and hot energy and transmitted it to the target using air convection. The cooling module was configured using the evaporative cooling system, and the heating module was configured using a coil heater (Figure 1). In this study, we set up the device to be able to transmit 15°C (cold), 41°C (hot), and alternating cold and hot temperatures to the target area.

### 3.2. Effect of Thermal Therapy on Ear-Swelling Response and Morphologic Change of TMA-Induced CHS Mice

To investigate whether treatment with different air temperature treatment can suppress the changes in ear phenotype induced by TMA, ear-swelling response and morphology of the ear were observed. As shown in Figure 2(b), the ear swelling rapidly increased in the TMA-induced CHS model compared to vehicle-treated mice and was further enhanced by hot thermal therapy. Moreover, symptoms including edema, erythema, and erosion were detected in TMA-induced CHS mice and markedly enhanced by hot thermal therapy or alternating cold/hot thermal therapy. However, it was markedly reversed with cold thermal therapy, and these alterations were significantly alleviated by treatment with cold thermal therapy (Figures 2(b) and 2(c)). Overall, these results clearly indicated that only cold thermal therapy may effectively protect the TMA-induced CHS; high or alternating cold/hot thermal therapies may have deficits in regard to CHS.

### 3.3. Effect of Thermal Therapy on PCA Reaction

**Figure 3:**
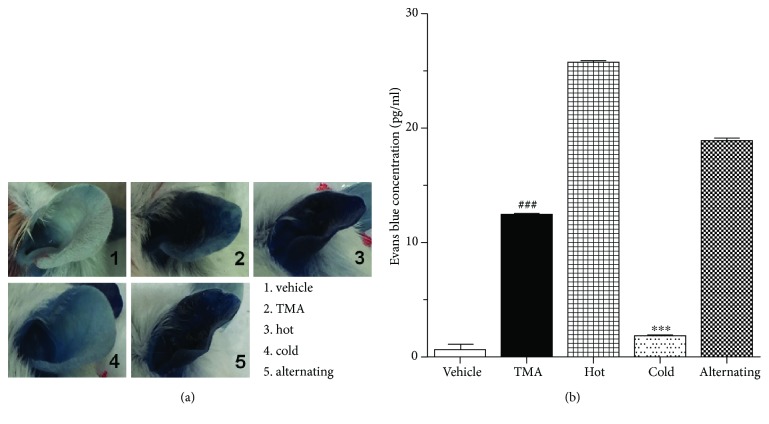
Effect of thermal therapy on passive cutaneous anaphylaxis of TMA-induced CHS. (a) Vascular permeability and (b) Evans blue concentrations were determined after the last TMA challenges. Data are shown as the mean ± SEM. Statistical comparisons were performed using one-way ANOVA, followed by the Fisher test. Significant differences at ^###^*P* < 0.001 compared with the vehicle group. ^∗∗∗^*P* < 0.001 compared with the TMA-induced CHS group.

The augment of vascular permeability of ear tissue is also a typical characteristic in an allergic inflammation model. In this study, the vascular permeability of ear tissue was measured to evaluate the effect of thermal energy therapy on TMA-induced CHS mice induced by tail vein injection of 0.5% Evans blue. As shown in Figure 3(a), 10 min after the last TMA challenge, there were obvious increases, becoming progressively darker blue in the left ears of TMA-induced CHS mice compared to vehicle-treated mice. PCA of hot and alternating cold/hot thermal therapies showed similar effects with that of TMA-induced CHS mice. However, the ears treated with cold thermal therapies remain light blue and white. These results were further confirmed by EBD concentration.  Figure 3(b) revealed that the TMA-induced CHS mouse ear had significantly elevated EBD concentration levels, which was reversed following treatment with cold thermal therapy. During the PCA reaction in mice, cold thermal therapy significantly inhibited vascular permeability.

### 3.4. Effect of Thermal Therapy on IgE Concentration in Serum

**Figure 4:**
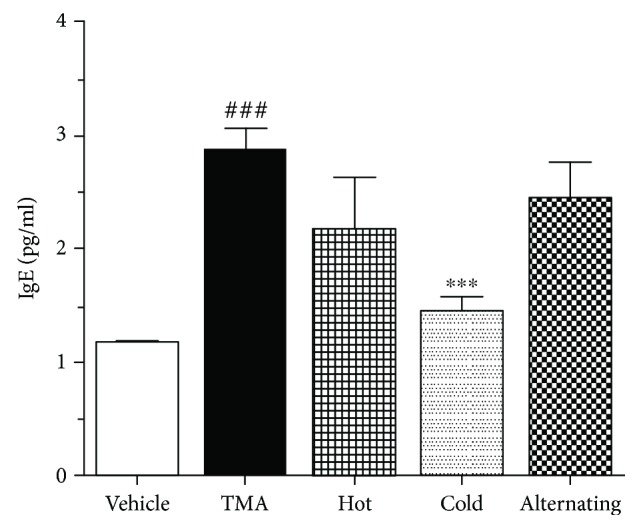
Effect of thermal therapy on the increased IgE of TMA-induced CHS. Serum was collected 24 hours after the last TMA challenge. Data are shown as the mean ± SEM. Statistical comparisons were performed using one-way ANOVA, followed by the Fisher test. Significant differences at ^###^*P* < 0.001 compared with the vehicle group. ^∗^*P* < 0.01 and ^∗∗^*P* < 0.05 compared with the TMA-induced CHS group.

Serum IgE is enhanced in most AD patients (Hoffmann et al., 1975); the ear tissue homogenate IgE concentration in the blood determined whether thermal therapy suppressed the allergic responses in TMA-induced CHS mice. TMA-challenged mice induced a significant increase in serum IgE concentration. In contrast, a strong decrease of IgE concentration was observed in the mice with cold thermal therapy (Figure 4). These results demonstrated that cold thermal therapy may effectively inhibit TMA-induced CHS.

### 3.5. Effect of Thermal Therapy on Inflammatory Responses in the Dermis of the Ear

**Figure 5:**
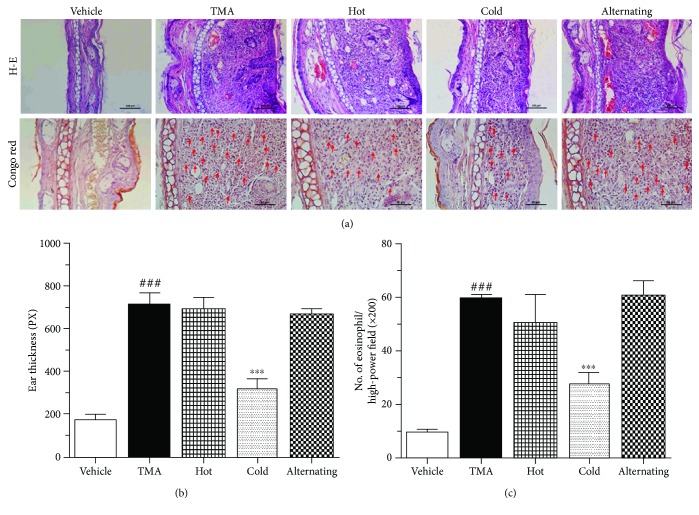
Effect of thermal therapy on histological changes of the ear in TMA-induced CHS. (a) Photographs of the ear stained by H-E and Congo red. (b) Ear thickness was taken 24 hours after the last TMA challenge. (c) The number of eosinophils in the ear was taken 24 hours after the last TMA challenge. Data are shown as the mean ± SEM. Statistical comparisons were performed using one-way ANOVA, followed by the Fisher test. Significant differences at ^###^*P* < 0.001 compared with the vehicle group. ^∗∗∗^*P* < 0.001 compared with the TMA-induced CHS group. Bar sizes are 50 *μ*m.

To evaluate the anti-inflammatory effect of air treatment on ear tissue, histological analysis of the ear was performed by HE staining. Prominent edema and increased inflammatory cell infiltration in the epidermis and dermis of the ear tissue were observed in TMA-induced CHS and hot thermal therapy mice (Figures 5(a) and 5(b)). However, the ear swelling was markedly decreased in the mice of thermal therapy, especially in the cold thermal therapy (Figures 5(a) and 5(b)). In addition, the vehicle-treated mice showed no significantly different effect.

### 3.6. Effect of Thermal Therapy on Eosinophil Responses in the Dermis of the Ear

Consistent with the experimental outcome mentioned above, the infiltration of eosinophils was measured as a cellular mechanism underlying the ear-swelling responses. The pathology pictures of mice ears stained with Congo red are presented in Figure 5(a). Compared with the vehicle group, TMA-induced CHS mice showed significant eosinophil infiltration in the dermis of the ear. Also, eosinophil infiltrations of hot and alternating cold/hot thermal therapies showed similar effects with that of TMA-induced CHS mice (Figures 5(a) and 5(c)). In contrast, cold thermal therapy markedly reduced the infiltration of eosinophils (Figures 5(a) and 5(c)). Collectively, these results suggest that cold thermal therapy attenuates TMA-induced CHS by blocking infiltration of eosinophil into the inflamed site.

### 3.7. Effect of Thermal Therapy on Mast Responses in the Dermis of the Ear

**Figure 6:**
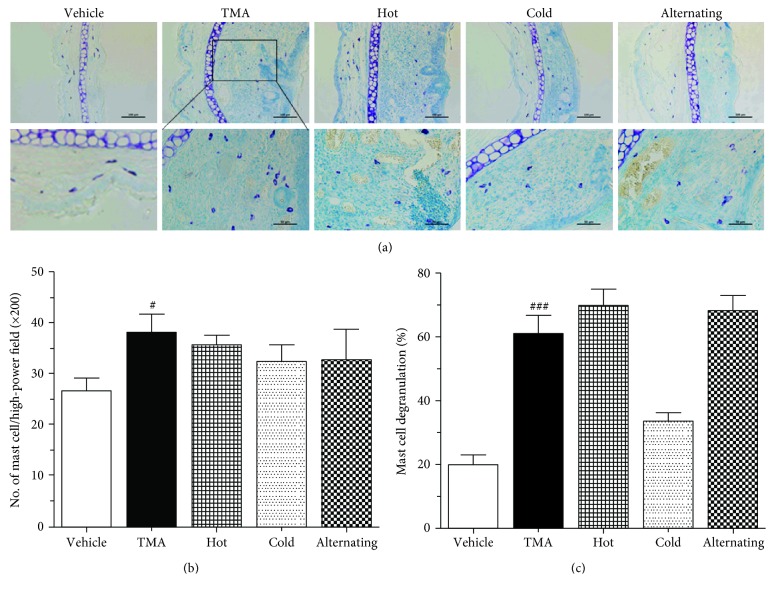
Effect of thermal therapy on mast cell activation in the ear of TMA-induced CHS. (a) Photographs of the toluidine blue-stained ear, (b) mast cell number in the dermis of the ear, and (c) mast cell degranulation rate were taken 24 hours after the last TMA challenge. Data are shown as the mean ± SEM. Statistical comparisons were performed using one-way ANOVA, followed by the Fisher test. Significant differences at ^###^*P* < 0.001 compared with the vehicle group. ^∗^*P* < 0.01, ^∗∗^*P* < 0.05, and ^∗∗∗^*P* < 0.001 compared with the TMA-induced CHS group. Bar sizes are 100 and 50 *μ*m.

As shown in Figure 6, the TMA challenge showed a tendency to increase slightly the mast cells' infiltration into the dermis of the ear in TMA-induced CHS mice. Moreover, the TMA challenge showed extensive degranulation processes and multiple granules extruding from mast cells, and the rate of mast cell degranulation was increased. Also, we revealed that hot or alternating cold/hot thermal therapy markedly induced the mast cell degranulation similar to that of TMA-induced CHS mice. However, cold thermal therapy suppressed mast cell infiltration and degranulation in the dermis of the ear (Figure 6). These results clearly indicated that cold thermal therapy attenuated skin inflammation resulting from the inhibition of the mast cell activation such as mast cell degranulation in the ear of TMA-induced CHS.

### 3.8. Effect of Thermal Therapy on the Release of Th2 Cytokines in Ear Homogenates

**Figure 7:**
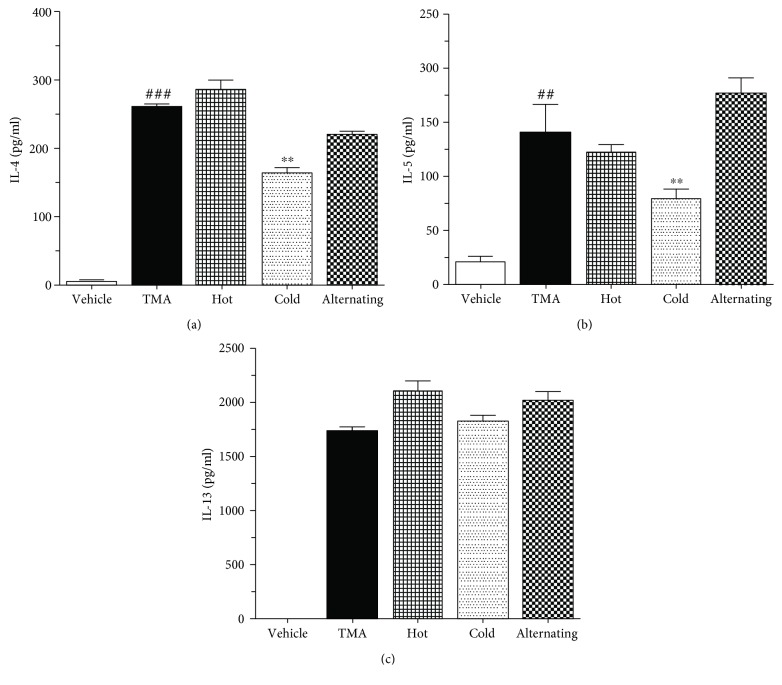
Effect of thermal therapy on Th2 cytokines in ear homogenates of TMA-induced CHS. (a) IL-4, (b) IL-5, and (c) IL-13 were taken 24 hours after the last TMA challenge. Data are shown as the mean ± SEM. Statistical comparisons were performed using one-way ANOVA, followed by the Fisher test. Significant differences at ^##^*P* < 0.05 and ^###^*P* < 0.001 compared with the vehicle group. ^∗∗^*P* < 0.05 compared with the TMA-induced CHS group.

To clarify the mechanisms underlying the attenuation of TMA-induced CHS mice, we examined the production of inflammatory cytokines including IL-4, IL-5, and IL-13 in the ear tissue using an ELISA kit. The levels of the Th2-mediated cytokine including IL-4 (Figure 7(a)), IL-5 (Figure 7(b)), and IL-13 (Figure 7(c)) were highly augmented in the ear tissue of TMA-induced CHS mice. However, the cold thermal therapy significantly decreased only the levels of IL-4 (Figure 7(a)) and IL-5 (Figure 7(b)). In addition, the levels of only IL-4 and IL-5 in ear homogenates positively correlated with the levels of EBD exudation in TMA-induced mice (Figure 7).

## 4. Discussion

In this study, a comprehensive study was performed to investigate the effect of cold therapy on the skin inflammation of TMA-induced dermatitis-like CHS in a BALB/c mouse model in comparison with hot and alternating cold/hot thermal therapies. And the possible mechanisms underlying the cold thermal therapy-induced inhibitory effects on the allergic dermatitis were investigated. The inhibitory mechanism of cold therapy observed in this study markedly suggests that the cold thermal therapy demonstrates potent antiallergic effects resulting from the inhibition of mast cell degranulation and Th2 cytokine secretion, especially IL-4 and IL-5.

TMA is sensitizer that induces occupational asthma in humans [29] and is routinely used to trigger T-cell-dependent CHS reactions and Th2-mediated skin inflammation in mice [8, 30, 31]. Responses of TMA-induced CHS are the infiltration of eosinophils and mast cells as well as the increased secretion of Th2 cytokines. Therefore, the severity of inflammation could easily be evaluated using the measurement of ear-swelling response (ear thickness). In our study, cold thermal therapy not only inhibited TMA-induced ear thickness but also ameliorated the infiltration of eosinophils and mast cells in the inflamed ear. Allergic responses are mainly based on the production and effect of IgE antibodies, and other immunoglobulin classes such as IgG1 and IgG2a have been the focus of allergy research [30, 32]. In addition, an allergic response is triggered after allergen-specific IgE and Th2 cells recognize exposed allergens in the environment, and its inflammatory process is involved with many different inflammatory cells including eosinophils and mast cells, cytokines, and other regulatory molecules [33, 34]. Therefore, we investigated the levels of total IgE in serum from TMA-induced CHS mice. TMA-induced CHS mice showed highly expressed total IgE in serum. However, cold thermal therapy decreased the level of total IgE, which is a hall marker of the Th2 immune response. These findings imply that cold thermal therapy improves TMA-induced CHS symptoms by reducing the level of total IgE.

Angiogenesis and enhanced microvascular permeability are typical characteristics of a large number of inflammatory diseases [35]. Skin mast cells passively sensitized with antigen-specific IgE are activated upon antigen challenge to cause vascular permeability increase and cutaneous swelling [36]. Cold thermal therapy suppressed the increased vascular permeability of the ear from TMA-induced CHS mice, and this result was consistent with the decrease of total IgE level, mast cell infiltration into the inflamed ear, and mast cell degranulation. These data suggest that cold thermal therapy relieves vascular permeability increase by diminishing the IgE level increase and mast cell activation such as mast cell degranulation.

Infiltration of eosinophils and mast cells into tissues is one of the main characteristics of human allergic inflammation [37], and recruitment of eosinophils and mast cells during allergic inflammation is associated with cells expressing mRNA for IL-4 and IL-5 [38, 39]. It has been reported that there is an association between clinical severity and Th2 cell-mediated immune imbalance [40], and IL-4 are related to Th2 differentiation and regulated IgE synthesis [41]. Consistent with the reduction of the infiltration of mast cells and eosinophils into the dermis of the ear, the levels of IL-4 and IL-5 and Th2 cytokines in the ear homogenate were also decreased after cold thermal therapy. The inhibition of IL-4 and IL-5 cytokine activity may contribute to the antiallergic effect of cold thermal therapy.

## 5. Conclusion

In conclusion, we have demonstrated that cold thermal therapy alleviates allergic skin inflammation in a TMA-induced CHS mouse model and exerts its effect by negatively regulating skewed IL-4 and IL-5 responses and mast cell activation. These findings suggest that cold thermal therapy could be a useful modality for the treatment of contact dermatitis and mast cell-mediated allergic diseases.

## Data Availability

The data used to support the findings of this study are available from the corresponding author upon request.

## References

[1] Avena-Woods C. (2017). Overview of atopic dermatitis. *The American Journal of Managed Care*.

[2] Simon D., Braathen L. R., Simon H. U. (2004). Eosinophils and atopic dermatitis. *Allergy*.

[3] David Boothe W., Tarbox J. A., Tarbox M. B. (2017). Atopic dermatitis: pathophysiology. *Advances in Experimental Medicine and Biology*.

[4] Homey B., Steinhoff M., Ruzicka T., Leung D. Y. M. (2006). Cytokines and chemokines orchestrate atopic skin inflammation. *The Journal of Allergy and Clinical Immunology*.

[5] Kimber I., Basketter D. A., Gerberick G. F., Dearman R. J. (2002). Allergic contact dermatitis. *International Immunopharmacology*.

[6] Kostner L., Anzengruber F., Guillod C., Recher M., Schmid-Grendelmeier P., Navarini A. A. (2017). Allergic contact dermatitis. *Immunology and Allergy Clinics of North America*.

[7] Dearman R. J., Basketter D. A., Kimber I. (1996). Characterization of chemical allergens as a function of divergent cytokine secretion profiles induced in mice. *Toxicology and Applied Pharmacology*.

[8] Dearman R. J., Warbrick E. V., Skinner R., Kimber I. (2002). Cytokine fingerprinting of chemical allergens: species comparisons and statistical analyses. *Food and Chemical Toxicology*.

[9] Mori T., Kabashima K., Yoshiki R. (2008). Cutaneous hypersensitivities to hapten are controlled by IFN-*γ*-upregulated keratinocyte Th1 chemokines and IFN-*γ*-downregulated langerhans cell Th2 chemokines. *The Journal of Investigative Dermatology*.

[10] Szabo S. J., Sullivan B. M., Peng S. L., Glimcher L. H. (2003). Molecular mechanisms regulating Th1 immune responses. *Annual Review of Immunology*.

[11] Shin J. U., Kim S. H., Noh J. Y. (2018). Allergen-specific immunotherapy induces regulatory T cells in an atopic dermatitis mouse model. *Allergy*.

[12] Wang B., Esche C., Mamelak A., Freed I., Watanabe H., Sauder D. N. (2003). Cytokine knockouts in contact hypersensitivity research. *Cytokine & Growth Factor Reviews*.

[13] Chai O. H., Lee H. K., Lee Y. C. (2005). Roles of TNF-*α* and IgE in the late phase of contact hypersensitivity induced by trimellitic anhydride. *Experimental & Molecular Medicine*.

[14] Vandebriel R. J., De Jong W. H., Spiekstra S. W. (2000). Assessment of preferential T-helper 1 or T-helper 2 induction by low molecular weight compounds using the local lymph node assay in conjunction with RT-PCR and ELISA for interferon-*γ* and interleukin-4. *Toxicology and Applied Pharmacology*.

[15] Dvorak H. F., Mihm MC Jr, Dvorak A. M. (1974). Morphology of delayed type hypersensitivity reactions in man. I. Quantitative description of the inflammatory response. *Laboratory Investigation*.

[16] Dvorak H. F., Mihm M. C., Dvorak A. M. (1976). Morphology of delayed-type hypersensitivity reactions in man. *The Journal of Investigative Dermatology*.

[17] Dvorak A. M., Mihm MC Jr, Dvorak H. F. (1976). Morphology of delayed-type hypersensitivity reactions in man. II. Ultrastructural alterations affecting the microvasculature and the tissue mast cells. *Laboratory Investigation*.

[18] Sugawara Y., Okamoto Y., Sawahata T., Tanaka K. (1993). Skin reactivity in guinea pigs sensitized with 2,4-toluene diisocyanate. *International Archives of Allergy and Immunology*.

[19] Diederich C. J. (2005). Thermal ablation and high-temperature thermal therapy: overview of technology and clinical implementation. *International Journal of Hyperthermia*.

[20] Sweeney E. E., Cano-Mejia J., Fernandes R. (2018). Photothermal therapy generates a thermal window of immunogenic cell death in neuroblastoma. *Small*.

[21] Tepperman P. S., Devlin M. (1986). The therapeutic use of local heat and cold. *Canadian Family Physician*.

[22] Imamura M., Biro S., Kihara T. (2001). Repeated thermal therapy improves impaired vascular endothelial function in patients with coronary risk factors. *Journal of the American College of Cardiology*.

[23] Beever R. (2010). The effects of repeated thermal therapy on quality of life in patients with type II diabetes mellitus. *Journal of Alternative and Complementary Medicine*.

[24] Zhu J., Zhang Y., Zhang A., He K., Liu P., Xu L. X. (2016). Cryo-thermal therapy elicits potent anti-tumor immunity by inducing extracellular Hsp70-dependent MDSC differentiation. *Scientific Reports*.

[25] Nadler S. F., Weingand K., Kruse R. J. (2004). The physiologic basis and clinical applications of cryotherapy and thermotherapy for the pain practitioner. *Pain Physician*.

[26] Habash R. W. Y., Bansal R., Krewski D., Alhafid H. T. (2006). Thermal therapy, part 1: an introduction to thermal therapy. *Critical Reviews in Biomedical Engineering*.

[27] Cameron M. H. (2014). *Physical Agents in Rehabilitation: from Research to Practice*.

[28] Lawrence J. C., Bull J. P. (2016). Thermal conditions which cause skin burns. *Engineering in Medicine*.

[29] Mapp C., Boschetto P., Miotto D., De Rosa E., Fabbri L. M. (1999). Mechanisms of occupational asthma. *Annals of Allergy, Asthma & Immunology*.

[30] Zhang X. D., Murray D. K., Lewis D. M., Siegel P. D. (2002). Dose-response and time course of specific IgE and IgG after single and repeated topical skin exposure to dry trimellitic anhydride powder in a Brown Norway rat model. *Allergy*.

[31] Schneider C., Döcke W.-D. F., Zollner T. M., Röse L. (2009). Chronic mouse model of TMA-induced contact hypersensitivity. *The Journal of Investigative Dermatology*.

[32] Hofmaier S., Comberiati P., Matricardi P. M. (2014). Immunoglobulin G in IgE-mediated allergy and allergen-specific immunotherapy. *European Annals of Allergy and Clinical Immunology*.

[33] Sin B., Togias A. (2011). Pathophysiology of allergic and nonallergic rhinitis. *Proceedings of the American Thoracic Society*.

[34] Minai-Fleminger Y., Levi-Schaffer F. (2009). Mast cells and eosinophils: the two key effector cells in allergic inflammation. *Inflammation Research*.

[35] Lange-Asschenfeldt B., Weninger W., Velasco P. (2002). Increased and prolonged inflammation and angiogenesis in delayed-type hypersensitivity reactions elicited in the skin of thrombospondin-2--deficient mice. *Blood*.

[36] Inagaki N., Nagai H. (2009). Analysis of the mechanism for the development of allergic skin inflammation and the application for its treatment:mouse models for the development of remedies for human allergic dermatitis. *Journal of Pharmacological Sciences*.

[37] Garcia G. (2006). Allergy-related hypereosinophilia. *Presse Médicale*.

[38] Nouri-Aria K. T., O'Brien F., Noble W., Jabcobson M. R., Rajakulasingam K., Durham S. R. (2000). Cytokine expression during allergen-induced late nasal responses: IL-4 and IL-5 mRNA is expressed early (at 6 h) predominantly by eosinophils. *Clinical and Experimental Allergy*.

[39] Wilson S. J., Shute J. K., Holgate S. T., Howarth P. H., Bradding P. (2000). Localization of interleukin (IL) -4 but not IL-5 to human mast cell secretory granules by immunoelectron microscopy. *Clinical and Experimental Allergy*.

[40] Sun R., Tang X. Y., Yang Y. (2016). Immune imbalance of regulatory T/type 2 helper cells in the pathogenesis of allergic rhinitis in children. *The Journal of Laryngology and Otology*.

[41] Cosmi L., Maggi L., Santarlasci V., Liotta F., Annunziato F. (2014). T helper cells plasticity in inflammation. *Cytometry. Part A*.

